# Influenza epidemiology and risk factors for severe acute respiratory infection in Morocco during the 2016/2017 and 2017/2018 seasons

**DOI:** 10.11604/pamj.2020.36.159.21239

**Published:** 2020-07-07

**Authors:** Hind Ezzine, Imad Cherkaoui, Ahmed Rguig, Hicham Oumzil, Mouad Mrabet, Abderrahman Bimouhen, Fatima El Falaki, Zakia Regragui, Zineb Tarhda, Mohammed Youbi, Mariam Naciri

**Affiliations:** 1 Directorate of Epidemiology and Disease Control, Ministry of Health, Morocco,; 2 Research Center (BIOBIO), Laboratory of Biodiversity, Ecology and Genome, Faculty of Sciences, University Mohammed V of Rabat, Morocco,; 3 National Influenza Center, National Institute of Hygiene, Ministry of Health, Morocco

**Keywords:** Influenza positivity rate, influenza risk factor, influenza-like illness, severe acute respiratory infections, co-morbidity

## Abstract

**Introduction:**

in order to implement an influenza vaccination program for high-risk-groups in Morocco, as recommended by the World Health Organization, an epidemiological study indicating the influenza virus effect in the development of complicated influenza for subjects with co-morbidity was required. The present study aims to evaluate the risk factors for severe acute respiratory infections caused by influenza in risk groups.

**Methods:**

this research is based on the epidemiological and virological surveillance data of severe acute respiratory infections and influenza-like illness during the 2016/2017 and 2017/2018 seasons. It was realized using a retrospective series study with a descriptive and analytical purpose.

**Results:**

the over-recruitment of pediatric cases with a severe acute respiratory infection has been significantly rectified because cases of severe acute respiratory infections under 15 years old in the 2017/2018 season represent only 57.9%, whereas they represented 75.9% of the total cases of severe acute respiratory infections during the 2016/2017 season. The influenza positivity rate has increased globally and specifically by age group, clinical service and co-morbidity. The risk factors considered were significantly associated with hospitalization for influenza-associated severe acute respiratory infections. The multivariate logistic regression analysis considers male sex (OR=2.1), age ≥65 years (OR=5.4), presence of influenza cases in the surroundings (OR=0.1), diabetes (OR=7.5) and chronic respiratory disease (OR=10.9) as risk factors influenza-associated severe acute respiratory infections.

**Conclusion:**

the risk assessment of influenza-associated severe acute respiratory infections in high-risk groups revealed national epidemiological findings, particularly for diabetics and the elderly. An influenza vaccination program for these high-risk-groups becomes much recommended in Morocco.

## Introduction

The seasonal influenza is an acute viral infection of the respiratory tract with high contagious effects. Both type A and B influenza viruses are responsible for seasonal epidemics of clinical influenza [1] which progress as Influenza-like illness (ILI), generally benign. Hence, in terms of morbidity, mortality and social costs, they impose a huge burden [2]. Hospitalization and deaths mainly occur in high-risk-groups due to severe acute respiratory infections (SARI). The seasonal epidemics affect about 9% of the world population each year [3] and cause about 3 to 5 million cases of serious diseases. Six hundred and fifty thousand deaths annually are associated with respiratory diseases caused by seasonal influenza [4]. Since the 1960s, the World Health Organization (WHO) has produced numerous resolutions and recommendations to take into account the morbidity and mortality burden of seasonal influenza epidemics. It also invited the member states to strengthen active influenza surveillance and vaccination programs, particularly for specific risk groups [5-8]. However, many countries around the world continue to have low immunization rates due to skepticism about the vaccine's efficacy [9-11]. The Moroccan sentinel system for epidemiological and virological surveillance of SARI and ILI was implemented in 2007 as part of the pandemic influenza response with the support of the Centers for Disease Control and Prevention of Atlanta, United States of America (US-CDC) [12].

Then, in 2014, it was redeployed in eight hospital and ambulatory sentinel sites. In 2013, the Moroccan Ministry of Health, responding to the recommendations of the WHO-SAGE committee concerning the influenza vaccination of risk groups [8], established a cooperation agreement with the US-CDC in order to extend the influenza vaccination to other risk groups in addition to health personnel. This project aims to conduct epidemiological and operational studies in order to demonstrate, to the national immunization technical advisory group (national technical and scientific advisory vaccination committee), the relevance of the influenza vaccination strategy [13]. Thus, the Moroccan Ministry of Health established in 2015 a prevention and control program for SARI and ILI, which expressed its information and monitoring needs. The evaluation of the surveillance system data collected during the 2015/2016 season revealed an over-recruitment bias in cases of pediatric SARI at sentinel hospital sites. 59.3% of the total cases of SARI recruited during this season are less than 4 years old [14]. Hence, many activities have been planned and carried out during the 2017/2018 season to rectify the recruitment bias observed at sentinel hospital sites in the previous season. In the present study, the results of epidemiological and virological surveillance of SARI and ILI in the 2016/2017 season were compared with those of the 2017/2018 season and the risk factors for influenza-associated SARI hospitalization were evaluated whatever the type of influenza viruses identified in confirmed influenza cases during the two seasons.

## Methods

**Type of study:** in this research, we have used a retrospective case series study with descriptive and analytical purpose.

**Study sample:** the case series includes cases of SARI and ILI collected as part of the epidemiological and virological surveillance that was implemented by the Moroccan Ministry of Health during the 2016/2017 and 2017/2018 seasons. The definition of ILI and SARI cases is the same as that used in the surveillance system which was updated in 2014 by the Ministry of Health [15] according to WHO's definitions [16].

**Severe acute respiratory infection:** this is a person suffering from an acute respiratory infection that includes: fever or a history of fever ≥ 38°C; cough; beginning of the disease in the last ten days; and which requires hospitalization. According to clinicians in sentinel hospitals, some patients may not develop a fever even if they have a viral infection due to metabolic and immune disorders. Therefore, some cases of SARI with co-morbidity were considered for recruitment purposes as meeting the case definition despite the absence of fever.

**Cases of ILI:** it is an acute respiratory infection that includes: measured fever ≥ 38°C; cough; and the beginning of the disease in the last ten days.

**Study sites:** eight public ambulatory and hospital sentinel sites: Rabat, Tangiers, Fez, Marrakech, Meknes, Beni Mellal, Oujda and Agadir and the private ambulatory network of medical practitioners were included in this study.

**Data collection:** the cases of SARI were collected by realizing a nasal and pharyngeal sampling immediately after admission to pediatric, pulmonary, medical and emergency wards or intensive care units as well as to the maternity wards of the sentinel hospital sites. The cases of ILI were collected in sentinel health centers. A questionnaire was successfully completed with patient identification data, clinical data, epidemiological data including vaccination, notion of travel and similar cases in the surroundings; results of virological analysis and information on the patient's evolution, particularly his death during hospitalization.

**Sample analysis:** the samples were sent to the National Influenza Center at the National Institute of Hygiene (CNR-INH) and analyzed on the same day using the real-time polymerase chain reaction (RT-PCR) method. A dynamic web application has been implemented for entering data from questionnaires of SARI and ILI cases, managing these data and reporting results back to sentinel sites.

**Statistical analysis:** data collected from the questionnaires of SARI and ILI cases were extracted as an Excel file from the web application. The statistical analysis was carried out using the Epi-Info 7 and SPSS software in 2 phases: 1°) a descriptive phase of epidemiological and virological surveillance data of SARI and ILI cases; 2°) an analytical phase (bivariate and multivariate analysis by logistic regression) in order to identify the risk factors of influenza-associated SARI whatever the type of influenza viruses identified. Only the cases of influenza confirmed by the laboratory during the two influenza seasons 2016/2017 and 2017/2018 were included in this analysis. The variable of interest in this study was the hospitalization due to the development of an influenza-associated SARI. The explicative variables studied were the potential risk factors related to personal socio-demographic characteristics, epidemiological context and the presence of co-morbidity. The statistical significance level was fixed at 5%.

## Results

**Figure 1:**
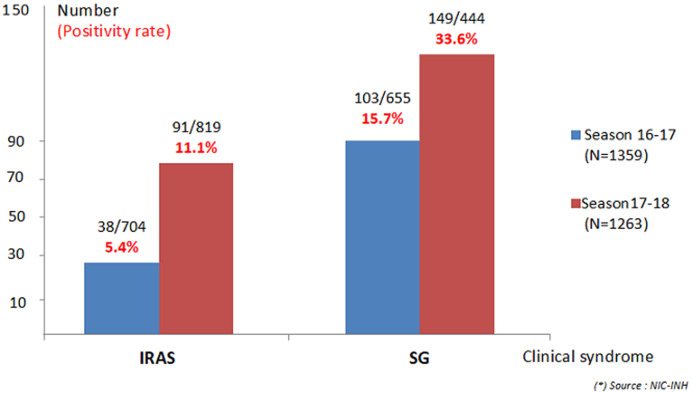
influenza positivity rate by clinical syndrome (2016/2017 and 2017/2018 season)

**Figure 2:**
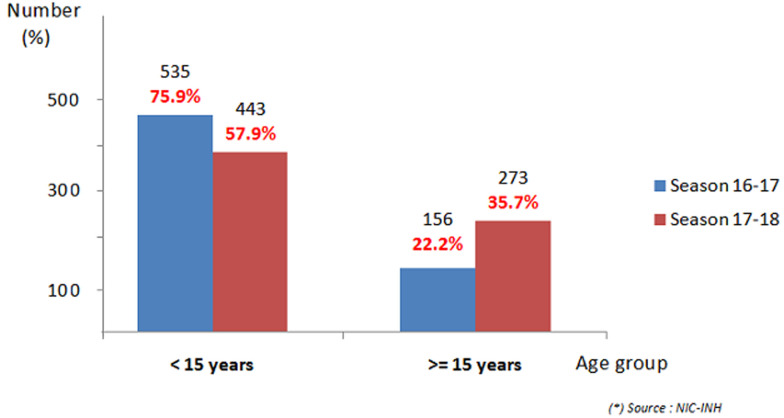
SARI cases per age range (2016/2017 and 2017/2018 season)

A total of 2622 patients were recruited during the two seasons. In 2016/2017 season, 1359 samples were collected and 1263 samples in 2017/2018 season (Figure 1). The proportion of SARI increased from one season to the other from 51.8% (n=704) to 64.8% (n=819). As shown in Figure 2, the proportion of SARI under 15 years old in the 2017/2018 season was 57.9%, while it represented 75.9% of the total number of SARI recruited during the 2016/2017 season. In Table 1, it is noted that the increase in recruitment of non-pediatric SARI cases has mainly concerned pulmonology, medicine and emergency wards as well as intensive care units. Similarly, the recruitment of SARI with co-morbidity has also increased from one season to the other, as shown in Table 2. According to the case definition used for recruitment, fever, cough and the beginning of signs within ten days were found in respectively 89.1%, 91.5% and 90.7% of SARI cases and in respectively 98.0%, 95.6% and 98.0% of ILI cases when both seasons’ cases were considered. The overall positivity rate is statistically higher during the 2017/2018 season compared to the 2016/2017 season (respectively 20.1% versus 10.4%, p<10^-6^). This increase is also individually observed for ILI and SARI as illustrated in Figure 1. The specific positivity rate per co-morbidity has also increased significantly, reaching values higher than 6% of the global positivity during the 2017/2018 season, particularly for diabetes, asthma and chronic respiratory disease.

**Table 1:** number of SARI cases recruited and their influenza positivity rates per clinical ward (2016/2017 and 2017/2018 season)

Clinical service	2016-2017 season	2017-2018 season
Pediatrics	527	434
	0,4%	7,6%
Pulmonology	89	142
	3,4%	7,6%
Medicine	1	40
	0,0%	22,5%
Intensive Care Unit	66	87
	9,1%	20,7%
Emergencies	7	33
	0,0%	9,4%

(^*^) Source : NIC-INH

**Table 2:** number of SARI cases with co-morbidities sampled and their influenza positivity rate (2016/2017 and 2017/2018 season)

Risk factor (or comorbidity)		2016-2017 season	2017-2018 season	Both seasons
Diabetes	Number	19	56	75
	Risk factor proportion	2,7%	6,8%	4,9%
	Risk factor positivity rate	5,3%	32,6%	24,6%
Obesity	Number	10	9	19
	Risk factor proportion	1,4%	1,1%	1,2%
	Risk factor positivity rate	10,0%	37,5%	22,2%
Asthma	Number	39	68	107
	Risk factor proportion	5,5%	8,3%	7,0%
	Risk factor positivity rate	15,4%	11,9%	13,2%
Chronic Respiratory Disease	Number	40	75	115
	Risk factor proportion	5,7%	9,2%	7,6%
	Risk factor positivity rate	7,5%	18,3%	14,4%
Chronic Heart Disease	Number	24	35	59
	Risk factor proportion	3,4%	4,3%	3,9%
	Risk factor positivity rate	0,0%	32,3%	18,2%
Chronic Renal Failure	Number	6	23	29
	Risk factor proportion	0,9%	2,8%	1,9%
	Risk factor positivity rate	0,0%	27,3%	21,4%
Chronic Neurological Disease	Number	2	4	6
	Risk factor proportion	0,3%	0,5%	0,4%
	Risk factor positivity rate	0,0%	25,0%	16,7%
Chronic Hematological Disease	Number	13	13	26
	Risk factor proportion	1,8%	1,6%	1,7%
	Risk factor positivity rate	7,7%	25,0%	16,0%
Pregnancy (^**^)	Number	9	5	14
	Risk factor proportion	3,10%	1,80%	2,40%
	Risk factor positivity rate	11,1%	40,0%	21,4%

(^*^) Source : NIC-INH (^**^) Only females

**Figure 3:**
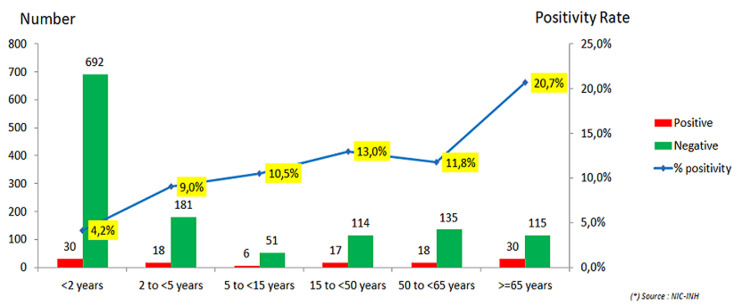
positive rate per age range of SARI cases, all-season combined (2016/2017 and 2017/2018 season)

**Figure 4:**
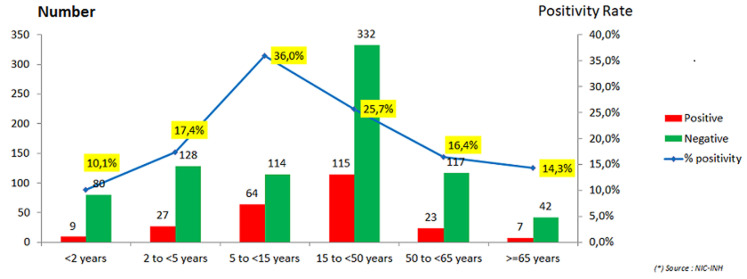
positive rate per age range of ILI cases, all seasons combined (2016/2017 and 2017/2018 season)

The participation of pregnant women remained very low during both seasons, as shown in Table 2, with recruitment of 9 and 5 pregnancy cases respectively. Figure 3and Figure 4illustrate that during the two seasons, the age range of 65 years and over in SARI cases and 5-15 years in ILI cases had the highest positivity rates with 20.7% and 36.0% respectively. The analysis of hospitalization risk factors for influenza-associated SARI was conducted on a sample of 381 influenza positive cases whatever clinical syndrome during the two seasons (except for pregnancy where only 198 female cases were considered). The risk factors considered in the bivariate analysis as shown in Table 3 and found significantly associated with hospitalization caused by influenza-associated SARI, included male sex (OR=2.2), age >65 years (OR=10.6), presence of influenza cases in the surroundings (OR=0.1), diabetes (OR=8.8), asthma (OR=10.1), chronic respiratory disease (OR=17.7), chronic heart disease (OR=4.2), chronic renal failure (OR=12.2) and chronic hematological disease (OR indeterminate). The multivariate analysis by logistic regression after adjustment for all previous risk factors, retained in the final model the male sex (OR=2.1 with CI 95%=[1.3-3.5] and p=0.005), age ≥65 years (OR=5.4 with CI 95%=[1.9-15.6] and p=0.002), the presence of influenza cases in the surroundings (OR=0.1 with CI 95%=[0.05-0.3] and p<10^-3^), diabetes (OR=7.5 with CI 95%=[1.7-32.8] and p=0.007) and chronic respiratory disease (OR=10.9 with CI 95%=[2.0-58.4] and p=0.005).These results are summarized in Table 4.

**Table 3:** bi-variate analysis for evaluating the risk factors of influenza-associated SARI with a sample of 381 influenza-positive cases (2016/2017 and 2017/2018 season)

Variable		SARI Positive n (%)		ILI Positive n (%)		OR Crude	CI95%	P Value
Socio-demographic characteristics								
Male sex	Yes	79	(61.2%)	105	(41.7%)	2.2	[1.4-3.4]	<0.001
	No	50	(38.8%)	147	58.3%)			
Age > 65 years	Yes	30	(23.3%)	7	(2.8%)	10.6	[4.5-24.9]	<10-11
	No	99	(76.7%)	245	(97.2%)			
**Context**								
Influenza vaccination during the current season	Yes	2	(1.6%)	7	(2.8%)	0.6	[0.1-2.7]	0.36(*)
	No	127	(98.4%)	245	(97.2%)			
Existence of cases in the surroundings	Yes	9	(7.0%)	95	(37.3%)	0.1	[0.06-0.3]	<10-11
	No	120	(93.0%)	157	(62.3%)			
**Co-morbidities**								
Diabetes	Yes	16	(12.4%)	4	(1.6%)	8.8	[2.9-26.9]	<10-5
	No	113	(87.6%)	248	(98.4%)			
Asthma	Yes	14	(10.9%)	3	(1.2%)	10.1	[2.8-35.9]	<10-4
	No	115	(89.1%)	249	(98.8%)			
Chronic Respiratory Disease	Yes	16	(12.4%)	2	(0.8%)	17.7	[4.0-78.3]	<10-5
	No	113	(87.6%)	250	(99.2%)			
Chronic Heart Disease	Yes	10	(7.8%)	5	(2.0%)	4.2	[1.4-12.4]	0.006
	No	119	(92.2%)	247	(98.0%)			
Chronic Renal Failure	Yes	6	(4.7%)	1	(0.4%)	12.2	[1.5-102.8]	0.007(*)
	No	123	(95.3%)	251	(99.6%)			
Chronic Neurological Disease	Yes	1	(0.8%)	0	(0.0%)	Ind.	Ind.	0.33(*)
	No	128	(99.2%)	252	(100.0%)			
Chronic Hematological Disease	Yes	4	(3.1%)	0	(0.0%)	Ind.	Ind.	0.01(*)
	No	125	(96.9%)	252	(100.0%)			
Pregnancy	Yes	3	(5.9%)	2	(1.4%)	4.5	[0.7-27.9]	0.11(*)
	No	48	(94.1%)	145	(98.6%)			
Obesity	Yes	4	(3.1%)	2	(0.8%)	4.0	[0.7-22.1]	0.10 (*)
	No	125	(96.9%)	250	(99.2%)			

(^*^) Use of the exact Fisher test because of the too small number in cells. (^**^) Only females

**Table 4:** multivariate logistic regression model for evaluating the risk factors of influenza-associated SARI with a sample of 381 influenza-positive cases (2016/2017 and 2017/2018 season)

Variable	OR ajusted	CI 95%		P-Value
Male sex (Yes/No)	2.1	1.3	3.5	0.005
Age >=65 years (Yes/No)	5.4	1.9	15.6	0.002
Existence of cases in the surroundings (Yes/No)	0.1	0.05	0.3	<0.001
Diabetes (Yes/No)	7.5	1.7	32.8	0.007
Asthma (Yes/No)	2.9	0.7	12.2	0.15
Chronic Respiratory Disease (Yes/No)	10.9	2.0	58.4	0.005
Chronic Heart Disease (Yes/No)	2.3	0.5	10.5	0.29
Chronic Renal Failure (Yes/No)	8.2	0.8	79.7	0.07

## Discussion

Since 2006, in response to the pandemic risk, several international and governmental institutions, such as WHO and US-CDC, have invested in partnership with African countries to develop their epidemiological surveillance and laboratory diagnostic capacities in the field of influenza. In 2009, the African network for influenza surveillance and epidemiology (ANISE), involving more than 30 African countries, was deployed to generate and disseminate data on influenza morbidity and mortality in Africa [17]. Influenza surveillance data from 2006 to 2010 obtained in 15 African countries, including two North African countries, Morocco and Egypt [18], showed that 21.7% of ILI cases (5165/69860) and 10.1% of SARI cases (4427/43620) were tested positive for influenza [19]. Similar to our case series, influenza is therefore detected consistently with a higher proportion for ILI cases compared to SARI cases, which probably reflects the higher sensibility of the ILI case definition compared to the SARI case definition. However, these results are slightly higher than those reported by other resource-limited countries outside Africa such as Bangladesh where the positivity rate is only 10% for ILI cases and 6% for SARI cases [20] as well as Cambodia and Indonesia with similar results [21,22]. In the African multicenter study, the burden of respiratory diseases and influenza was highest among children less than 5 years of age, which is consistent with other studies conducted in the region [23,24].

The children are probably over-represented, which explains the low proportions of influenza infection in the older age range. The efforts made at the start and during the 2017/2018 influenza season to reactivate the sentinel system of epidemiological and virological surveillance in Morocco have successfully limited the recruitment bias of cases of pediatric, while the recruitment of ILI cases was adequately represented in each age range from one season to the other. The influenza positivity rate results obtained in 2017/2018 season, with 11.9% for SARI cases, were very close to the average of 15 African countries. However, the influenza positivity rate was 34.8% for ILI cases, which is much higher, indicating a better recruitment of ILI cases in Morocco. The risk assessment of influenza infection according to age group in the two seasons 2016/2017 and 2017/2018 has revealed that influenza infection in Morocco mainly affects SARI cases over the age of 65 with an influenza positivity rate of 20.7%.The influenza positivity rate in SARI cases under 2 years of age remains relatively low in Morocco during the study period with 4.4%, which is consistent with the results of international [25-28] and national [29] studies showing the preponderance of other viruses such as rhinovirus and RSV in children with bronchoalveolitis.

In contrast, the 5-15 age range has the highest influenza positivity rate in ILI cases, with 36.0%, probably due to school exposure. Despite the progress in the management of serious infectious diseases, community pneumonia, which is the major clinical presentation of SARI cases, remains the primary cause of hospitalization and death in developed countries [30, 31]. The influenza virus causes about 6.5% of all cases of pneumonia [32] and up to 64% of their viral cause [33]. Available studies on the impact of influenza virus infection on morbidity and mortality in low-income countries are particularly limited, although this impact may be amplified by specific factors such as overpopulation and inadequate access to health services. However, if the vast majority of patients have benign influenza during seasonal peaks, other patients with specific risk factors will develop a severe acute respiratory infection [34]. Hence, an increase in the number of cases of acute asthma [35], acute exacerbation of chronic obstructive pulmonary disease (COPD) [36], decompensating of metabolic diseases (diabetes) [37], cardiac diseases [38] or other [39-41] and an increase in hospitalizations for extreme ages (children and elderly people) [42] as well as pregnant women [43] are usually observed. This leads rapidly to an overload of admission capacities of emergency wards and intensive care units (ICUs) [44], where clinical management of this type of infection will depend mainly on the patient's condition rather than the virulence of the virus.

In bivariate analysis of our study for both seasons, the majority of co-morbidities recommended by WHO for influenza vaccination are strongly associated with the hospitalization for influenza-associated SARI whatever the type of virus identified (age ≥65 years, diabetes, asthma, chronic respiratory disease, chronic heart disease, chronic kidney failure and hematologic disease).The other factors such as pregnancy and neurological disease recruited in very small numbers did not have sufficient statistical power to assess their risk. Thus, the multivariate logistic regression model retains only three risk factors after adjustment, namely age ≥65 years, diabetes and chronic respiratory disease. The risk associated with chronic renal failure remains at the limit of statistical significance where p=7% whereas the risk of chronic heart disease is certainly the subject of a confounding effect due to the statistical association with the presence of diabetes. It should be noted that the last two diseases are often associated in current clinical practice. These risk factors are usually found associated with hospitalization for influenza-associated SARI cases in several studies carried out in both low-income and developed countries in particular for extremes of age, diabetes, chronic respiratory diseases as asthma and HIV when the prevalence is high [45-49]. In addition, male sex is statistically strongly associated with the hospitalization for influenza-associated SARI due to the over-recruitment of women with ILI at sentinel health centers. Moreover, the presence of influenza cases in the surroundings appears to have a protective effect on the risk of hospitalization for SARI due to the fact that ambulatory subjects recruited from sentinel health centers are more exposed to the dissemination of the virus than subjects with SARI recruited in a closed hospital environment.

## Conclusion

In this study, the analysis of epidemiological and virological sentinel surveillance data collected during the 2016/2017 and 2017/2018 seasons allowed us to assess the risk of hospitalization due to the occurrence of influenza-associated SARI. Consequently, this evaluation provides national epidemiological evidence, particularly for diabetics and elderly persons. As recommended by the WHO, an influenza vaccination program for these high-risk-groups is therefore highly appropriate in Morocco. Finally, in order to assess other risk factors, particularly pregnancy, further studies should be conducted with larger samples.

### What is known about this topic

- Following a comprehensive review of the influenza disease burden, vaccine performance and safety in populations of all ages and at-risk groups, incorporating available data from low and middle-income country settings, the WHO strategic advisory group of experts (SAGE) on immunization met on 10-12 April 2012 in Geneva, Switzerland and recommended pregnant women as the most important risk group for inactivated seasonal influenza vaccination. Other risk groups to be considered, in no specific priority order were: health-care workers; children aged 6-59 months; the elderly and those with high risk conditions (Reference: WER No. 21, 2012, 87, 201-216).

### What this study adds

- At the request of the Morocco national immunization technical advisory group (NITAG) and before the implementing of the Morocco influenza vaccination strategy targeting high risk groups as recommended by WHO, the influenza surveillance team carried out epidemiological studies based on data provided by the influenza surveillance system to produce the evidence in Morocco of the influenza vaccination benefit as done by WHO at international level.
